# Enamel Defects in Deciduous Dentition and Their Association with the Occurrence of Adverse Effects from Pregnancy to Early Childhood

**DOI:** 10.3290/j.ohpd.a45077

**Published:** 2020-09-04

**Authors:** Mário Batista Ciríaco Neto, Kassia Paloma da Silva-Souza, Valéria Fernandes Maranhão, Kátia Virginia Guerra Botelho, Mônica Vilela Heimer, Valdeci Elias Dos Santos-Junior

**Affiliations:** a Mário Batista Ciríaco Neto Dentist, Department of Paediatric Dentistry, Dental School, Integrated School of Pernambuco, Maceió, Alagoas, Brazil. Examined and diagnosed all participating children, read and approved the final manuscript.; b Kassia Paloma da Silva-Souza Dentist, Department of Paediatric Dentistry, Dental School, Integrated School of Pernambuco, Maceió, Alagoas, Brazil. Field work, data collection, read and approved the final manuscript.; c Valéria Fernandes Maranhão Adjunct Professor, Department of Paediatric Dentistry, Dental School, Integrated School of Pernambuco, Maceió, Alagoas, Brazil. Supervised study, study design, epidemiological data analysis, drafted the manuscript, read and approved the final manuscript.; d Kátia Virginia Guerra Botelho Associate Professor, Department of Paediatric Dentistry, Dental School, Integrated School of Pernambuco, Maceió, Alagoas, Brazil. Supervised study, guided study design, performed statistical analysis, drafted the manuscript, read and approved the final manuscript.; e Mônica Vilela Heimer Adjunct Professor, Department of Paediatric Dentistry, University of Pernambuco, Maceió, Alagoas, Brazil. Supervised study, study design, performed statistical analysis, drafted the manuscript read, approved the final manuscript.; f Valdeci Elias Dos Santos-Junior Adjunct Professor, Department of Paediatric Dentistry, Federal University of Alagoas, Maceió, Alagoas, Brazil. Read and approved the final manuscript.

**Keywords:** child, child health, dental enamel hypoplasia, pregnancy

## Abstract

**Purpose:**

To verify the prevalence of developmental defects of enamel (DDE) in deciduous teeth and analyse the association with adverse events that occurred during pregnancy and early childhood.

**Materials and Methods:**

In a cross-sectional study, 152 children with an average age of 3.57 ± 1.25 years were examined according to the criteria established by the DDE index. A previously validated questionnaire was given to mothers in order to obtain information regarding: calcium and vitamin D deficiency (measured in mothers); gestational diabetes; gestational undernutrition; weight at birth; neonatal hypoxia; and presence of asthma in early childhood. The clinical exam was conducted by a single examiner calibrated for visual exams (Kappa = 0.84), outdoors on patios of schools with children and examiner knee to knee. Pearson’s chi-squared test and Fisher’s Exact Test (p < 0.05) were used to determine statistically significant associations between the variables in study. The data were then analysed using a binary logistic regression regression.

**Results:**

26.3% of children exhibited DDE. It was possible to verify a statistically significant association between DDE and vitamin D deficiency (p < 0.01), calcium deficiency (p = 0.01), neonatal hypoxia (p = 0.026), and gestational diabetes (p = 0.04). The regression model allowed the conclusion that children who had neonatal hypoxia during childbirth, gestational diabetes or vitamin D deficiency during their gestation were 3.54, 12.47 and 6.40 more likely to exhibit signs of DDE, respectively.

**Conclusion:**

The prevalence of DDE was considered high and was associated with vitamin D and calcium deficiency during pregnancy, gestational diabetes, and neonatal hypoxia.

Tooth enamel can act as a biological marker, thanks to the lack of tissue remodelling throughout the life of an individual, with alterations occurring during its formation being permanently detectable.^12,21^ From this perspective, adverse events occurring prenatally, perinatally, and during birth may determine an individual’s oral health.^8^

The presence of developmental defects of enamel (DDE) in children may have negative impacts on the perception of their oral conditions, interfering in their quality of life.^22^ These alterations in tooth enamel may be accompanied by dental sensitivity, occlusal disease, and a higher susceptibility to premature caries in childhood.^6,19^ Furthermore, dysfunctions in the development of enamel in deciduous teeth may act as a predictive factor for similar alterations in permanent teeth.^4^

However, DDE’s aetiology is still not entirely clear, with the available evidence being sometimes contradictory.^14^ Studies on deciduous dentition are limited, and the literature still lacks evidence to explain the prevalence of such alterations and events occurring during pregnancy.^7^ The formation and maturation of deciduous teeth is susceptible to maternal metabolic imbalance. Excess glucose that characterises gestational diabetes,^3,26^ or even deficiency of calcium and vitamin D1,^24^ may affect the secretion of proteins that form the structure of dental enamel. Oxygen deficiency during childbirth has been reported as a possible metabolic disorder acting on odontoblasts, resulting in the formation of abnormal tissue.^2,9^

Thus, this epidemiological, cross-sectional study was aimed at verifying the association of adverse events occurring during pregnancy and early childhood with enamel defects in deciduous teeth in Brazilian children

## MATERIALS AND METHODS

### Ethics Statement

This cross-sectional study was carried out in the Brazilian city of Recife, state of Pernambuco. The study was approved by the Research Ethics Committee of the Integrated Faculty of Pernambuco (Protocol No. 2.263.398 / 2017) and followed the ethical and legal principles that regulate research on human beings, according to Resolution n. 466/2012 of the National Research Committee. This research was conducted in accordance with Declaration of Helsinki. Written information explaining the purpose of the study was sent to the parents who submitted the signed written consent. Verbal consent was obtained from children prior to clinical examination.

### Pilot Study and Sample Size 

To determine the sample size and validity of the methodological procedures, a pilot study was conducted that included 70 children from 2 to 5 years of age attending two state schools. The prevalence of DDE found in the pilot study was then used to calculate the sample required for the main investigation.

In order to carry out the sample calculations, epi-Info software (CDC; Atlanta, GA, USA) was used, considering a confidence interval (CI) of 95%, a standard error of 5% and the prevalence of DDE of 11.1%, derived from the pilot study. Hence, the final sample consisted of 152 children, with ages ranging from 2 to 5 years, and their respective mothers.

### Data Collection and Clinical Examination 

The children were randomly selected from 14 of 58 public nursery schools by stratified randomised sampling. The nursery schools were randomly selected from each administrative region in proportion to the total number of schools in each region.

The clinical examination was conducted by a single examiner calibrated for visual exams (Kappa = 0.84), following the criteria established by the Modified Developmental Dental Enamel Index (DDE) Index.^12^ The exams were performed on the patios of schools, under natural light, with children knee to knee with the examiner. Each dental element was cleaned and dried with sterile gauze and examined using odontoscopes.

A questionnaire, previously validated in the pilot study, was given to the mothers at the time when children left school. During the interview, information was collected regarding pre-natal exams (calcium and vitamin D deficiency), birth (prematurity, weight at birth, and neonatal hypoxia), and the children’s gender, age at the time of the exam, and the presence of asthma.

In order to characterise the variables related to weight at birth and prematurity, parameters established by the WHO were taken into account.^25^ Thus, children born before gestational week 37 were considered premature, and children born weighing < 2500 g were classified as underweight neonates. It is important to point out that during pregnancy, pregnant women receive several exams to monitor the development of the fetus, therefore making it possible to collect all variables of interest for the current study.

### Statistical Analysis

Data analysis and processing were carried out using the SPSS v 23 (SPSS/IBM; Armonk, NY, USA). In order to test the association between the two categorical variables, Pearson’s chi-squared and Fisher’s Exact tests were used. The normal distribution of data was verified with the Kolmogrov-Smirnov test. In order to verify which variables influence defects of enamel in deciduous teeth, a logistic regression model was adjusted with the variables that showed significant association up to 20% (p < 0.20) in the bivariate analysis. Through this model, it was possible to estimate the OR values and the respective p-values of the variables from each category in terms of the reference category. The error margin used in the decisions of the statistical tests was 5%, with a 95% CI.

## RESULTS

One hundred fifty-two children with an average age of 3.57 ± 1.25 years were examined. Most were girls (53.3%). The prevalence of enamel defects was 26.3%. Enamel hypoplasia was the most prevalent defect detected (75%), followed by demarcated opacity and diffuse opacity, both with a 12.5% prevalence. No simultaneous cases of opacity (neither demarcated nor diffuse) and hypoplasia were observed. The incisors were the most frequently affected teeth (23%), followed by the canines (9.9%) and molars (2%).

The description of the independent variables related to enamel defects is presented in Table 1. Statistically significant differences were found between the enamel defects and calcium deficiency (p = 0.01), vitamin D deficiency (p < 0.001), gestational diabetes (p < 0.04) and neonatal hypoxia (p < 0.026).

**Table 1:** Description of the independent variables associated with the presence of enamel defects in deciduous dentition

Variables	Defect present	
Yes	No	p-value
n (%)	n (%)	
**Calcium deficiency**			
Yes	15 (50.0)	15 (50.0)	p* = 0.001
No	25 (20.5)	97 (79.5)	
**Vitamin D deficiency**			
Yes	20 (54.1)	17 (45.9)	p* < 0.001
No	20 (17.4)	95 (82.6)	
**Child’s gender**			
Male	17 (23.9)	54 (76.1)	p* = 0.534
Female	23 (28.4)	58 (71.6)	
**Prematurity**			
Yes	2 (22.2)	7 (77.8)	p** = 1.000
No	38 (26.6)	105 (73.4)	
**Gestational diabetes**			
Yes	6 (75.0)	2 (25.0)	p** = 0.004
No	34 (23.6)	110 (76.4)	
**Gestational undernutrition**			
Yes	1 (33.3)	2 (66.7)	p** = 1.000
No	39 (26.2)	110 (73.8)	
**Neonatal hypoxia**			
Yes	8 (53.3)	7 (46.7)	p** = 0.026
No	32 (23.4)	105 (76.6)	
**Presence of asthma**			
Yes	5 (31.3)	11 (68.7)	p** = 0.764
No	35 (25.7)	101 (74.3)	
*Chi-squared test; **Fisher’s Exact Test. Statistical significance set at 5%.

To create the “enter” type binary logistic regression model, the following variables were inserted: calcium deficiency, vitamin D deficiency, prematurity, gestational diabetes, gestational undernutrition, neonatal hypoxia and the presence of asthma. Through this model, all explanatory variables entered the model at the same time. Those that did not remain statistically significant were excluded, yielding the final model. The order in which the variables left the model was: prematurity, calcium deficiency, presence of asthma, and finally, gestational malnutrition.

The collinearity condition was verified. Through the regression model, it was possible to conclude that children who had neonatal hypoxia during childbirth, gestational diabetes, or vitamin D deficiency during their gestation were 3.54, 12.47, and 6.40 times more likely to exhibit signs of DDE, respectively (Table 2).

**Table 2:** Results of binary logistic regression (enter type) for the prevalence of enamel defects

Variables	Odds	95% CI	p-value
**Vitamin D deficiency**			
Yes	6.40	2.71 – 15.12	<0.001
No	1.00	–	
**Neonatal hypoxia**			
Yes	3.54	1.02 – 12.25	0.046
No	1.00	–	
**Gestational diabetes**			
Yes	12.47	2.15-72.45	0.005
No	1	–	
**Prematurity**			
Yes	0.00	0.00	0.999
No	1	–	
**Calcium deficiency**			
Yes	0.94	0.23-3.77	0.934
No	1	–	
**Presence of asthma**			
Yes	0.53	0.12-2.29	0.395
No	1	–	
**Gestational undernutrition**			
Yes	0.02	0.00–1.19	0.061
No	1	–	


## DISCUSSION

The present study showed that pregnancies with a background of vitamin D deficiency, calcium deficiency, gestational diabetes and neonatal hypoxia are considered risk factors for the development of enamel defects in deciduous dentition of children. However, the mechanism and aetiological factors are not entirely known.^14^ It is important to point out that amelogenesis, the process of forming dental enamel, begins in the 15th week of intrauterine life. Most of it is formed during the prenatal stage, and the rest throughout childhood.^8,27^ This may explain the presence of enamel defects, since the risk factors occur throughout the same period.

Adequate serological levels of vitamin D (concentrations higher than 30 nmol L-1) tend to yield the best oral health conditions. During the prenatal stage, vitamin D deficiency has been associated with adverse effects, such as the delay in the eruption sequence, gum inflammation, and defects in enamel development.^13,28^ Opacities and/or hypoplasia in dental enamel are the most common defects regarding this type of deficiency.^20^ The most accepted explanation is linked to the malfunction of protein secretion of the enamel matrix and subsequent hypomineralisation.^21^ The findings from this research corroborate this explanaion, as the children whose mothers suffered from vitamin D deficiency during pregnancy were approximately 9 times more likely to have DDE.

Enamel hypoplasia has been reported as the main type of enamel defect when calcium levels are insufficient during maturation of the mineral content.^20,21^ This type of defect was also the most prevalent finding in this study (75%). Due to the early mineralisation of deciduous incisors, such data suggests that a vitamin D and calcium deficiency during pregnancy has a significant influence on the etiology of this defect.^1,24^

Neonatal hypoxia is a result of oxygen deprivation during birth. The reduced oxygen levels may lead to disorders of dental enamel matrix secretion, due to the detrimental influence on the cellular metabolism of ameloblasts.^9^ The results of the present study corroborate those of the study by Arrow,^2^ which reports that such deficiencies occurring during the first years of life expose children to a greater risk of developing enamel defects. Furthermore, the current study indicated that in the case of neonatal hypoxia, the children were nearly seven times more likely to have DDE.

Gestational diabetes mellitus (GDM) is an endocrine disorder defined as an alteration of carbohydrate metabolism leading to hyperglycaemia of varying severity, first diagnosed or beginning during pregnancy, which may or may not persist until after birth.^3^ It is the most common metabolic problem experienced during pregnancy and occurs in 3% to 13% of pregancies.^26^ In the present study, 5.3% of the mothers interviewed suffered from gestational diabetes. A statistically significant association between this health condition during pregnancy and the presence of enamel defects in deciduous dentition was verified. To explain this finding, it is important to observe that the biochemical disorders occur due to the excess of glucose, which lead to a malfunction of the ameoblasts.^3,26^ No statistically significant association was noted between prematurity and enamel defects. Although the literature^15,16^ commonly reports such an association, the paradoxical result in the current study may have occurred due to the size of the sample or even as a result of population variability. Therefore, the results obtained must be interpreted with caution. Some studies^11,23^ still state that asthma in early childhood is considered a risk factor for the development of enamel defects. However, this was not confirmed by the present research.

The presence of hypoplasia was verified as the most prevalent defect (75%), followed by diffuse and demarcated opacities (25%). Nonetheless, the literature^12^ has shown that opacities are the most prevalent type of enamel defect in deciduous dentition. Data analysis and risk factor verification in the present work (vitamin D deficiency, calcium deficiency, neonatal hypoxia and gestational diabetes) showed that early exposure to adverse events predisposes toward hypoplasia, a structural disorder related to the enamel matrix protein. The incisors were the most commonly affected teeth (23%), followed by the canines (9.9%) and molars (2%). This variation may be related to the different times at which the dental elements form. Thus, the formation and maturation of enamel in the different elements may be taken as a predictive factor to clarify when the adverse event occurred; that is, this tissue may act as a biomarker.^12,17^

Besides the aesthetic issue of enamel defects, according to a systematic review,^5^ these anomalies predispose to early childhood caries. The authors argue that this is due to bacterial adherence and colonisation on the surface of the most porous enamel areas, intensifying the cariogenic challenge. Thus, dental health is still modulated by developmental and maturation stages of dental tissues, with environmental factors exerting a significant influence.

## CONCLUSION

Approximately one-fourth of the children examined exhibited at least one tooth with an enamel defect. Calcium and vitamin D deficiencies, gestational diabetes and neonatal hypoxia are thus considered risk factors for the development of the dental enamel defects in deciduous teeth.

## REFERECES
